# Effectiveness of Physical Therapy in a Comminuted Patella Fracture Managed with Tension Band Wiring: A Case Report

**DOI:** 10.7759/cureus.50870

**Published:** 2023-12-20

**Authors:** Divya R Jain, Mitushi Deshmukh

**Affiliations:** 1 Department of Musculoskeletal Physiotherapy, Ravi Nair Physiotherapy College, Datta Meghe Institute of Higher Education and Research, Wardha, IND

**Keywords:** rehabilitation, physiotherapy, isometric exercises, tension band wiring, patellar fracture

## Abstract

A patella fracture occurs when the patella bone, which covers the knee joint, breaks. A severe injury, such as a fall or a hit to the patella, is frequently the cause. There are two types of patella fractures: basic and complicated. The treatment of certain fractures necessitates surgery. Patella fracture symptoms include pain, swelling, bruising, inability to straighten the leg, and inability to walk. Rehabilitation aims to increase the range of motion, increase muscles’ strength, and make the patient functionally independent. We report the case of a 69-year-old female with a comminuted patella fracture managed with open reduction and internal fixation (ORIF) with tension band wiring. A four-week inpatient rehabilitation increasing range of motion and improving strength has shown a tremendous improvement in the patient’s symptoms.

## Introduction

Patella fractures are rather frequent, accounting for around 1% of all fractures. Surgery is commonly used for reconstructing displaced patella fractures or those affecting the extensor mechanism. Tension band wire (TBW) construction with cerclage wiring or TBW through cannulated screws as an option is the current standard. "Difficult patella fractures" are those in which the osteopenic bone cannot sustain a TBW and/or cerclage, leading to fixation failure prior to the bone union in elderly patients, particularly those with comminuted patella fractures [1]. This approach has been demonstrated to be quite effective in the treatment of fractures, but it is also linked with hardware-related problems such as wire prominence, skin irritation, infection, hardware migration, and hardware breakage. In cadaveric and experimental trials, a braided polyester non-absorbable suture, rather than a stainless steel cerclage wire, was used to build the principle tension-band construct, with reported good clinical outcomes, fracture union, and decreased complication rates [2].

Direct or indirect forces can cause patellar fractures. The majority of them are caused by direct injuries to the patella, such as a fall, a car accident, or a combination of these. In high-energy traumas, the patient with a patellar fracture should be checked for hip dislocations, ipsilateral femoral neck or shaft fractures, distal femur fractures, and proximal tibia fractures. A near-fall, a fall from a height, or a compounded accident might cause indirect damage [3]. It's critical to have a systemic assessment of all the limbs when dealing with trauma patients so that the dimension, position, and degree of soft tissue involvement may be documented before reduction or splinting. Many studies have claimed that patellectomy after comminuted patellar fractures improves rehabilitation outcomes; however, subsequent investigations have found that preserving the patella for better outcomes in comminuted open patella fractures is preferable. Fibrosis is a typical cause of loss of knee mobility after a patellar fracture. Cyriax's cross-friction massage therapy for fibrosis and scar tissue is now the most commonly approved in clinical practice [4].

Passive range of motion (ROM) exercises can begin 48 hours following surgery with stable fixation, according to Koval and Kim [5]. After that, patients can go on to active ROM and isometric activities. Progressive resistance workouts are added after there is radiographic evidence of recovery. Lower extremity fractures are one of the most prevalent disorders that orthopaedic surgeons treat, and providing adequate weight-bearing recommendations is a critical clinical problem. Early weight-bearing may increase function and speed in the return to work process, lowering the cost of an accident. Allowing patients to bear weight too quickly, on the other hand, may result in loss of reduction or fixation failure, jeopardizing patient outcomes and perhaps demanding additional surgical intervention [6]. Here, we report a case of comminuted patellar fracture with tension band wiring and the efficacy of a systematized rehabilitation program in the same.

## Case presentation

**Figure 1:**
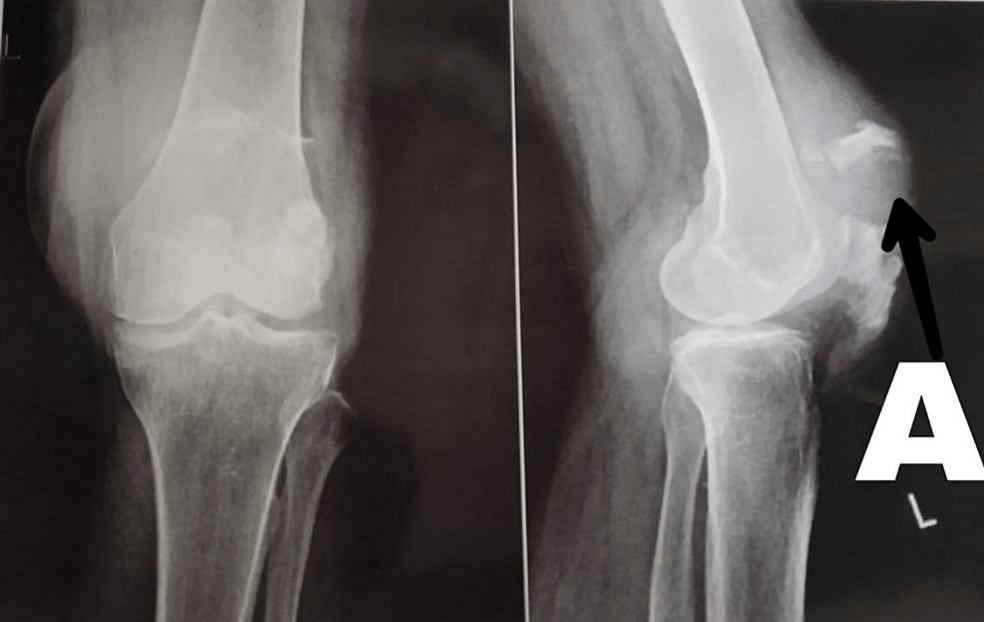
Preoperative X-ray of comminuted patella fracture

**Figure 2:**
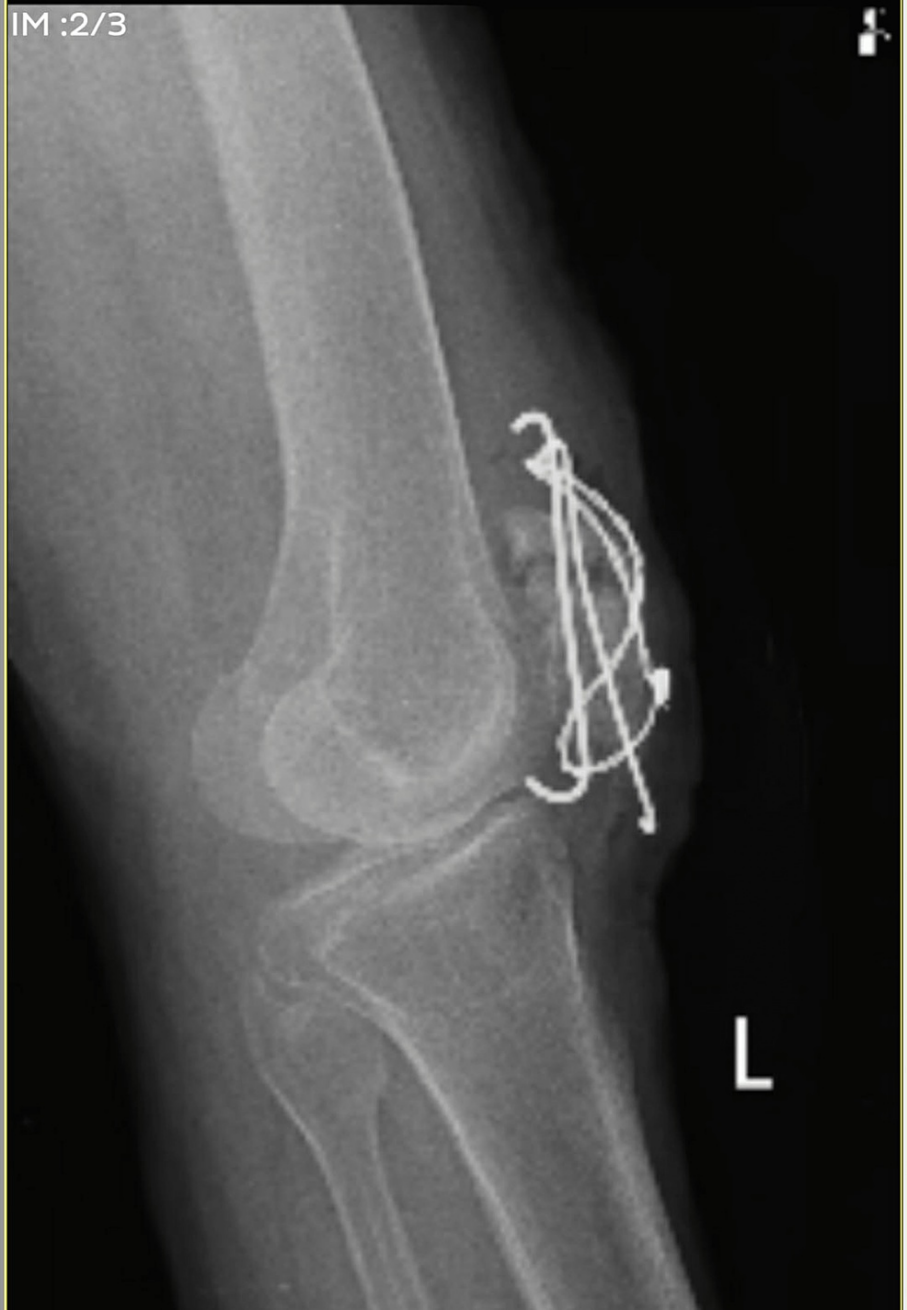
Postoperative X-ray of comminuted patellar fracture surgically treated with tension band wiring.

A 69-year-old female, with a mesomorphic built and right-hand dominance, slipped slip and fell in the bathroom a week ago, sustaining an injury to the left knee. The patient was otherwise healthy at the time. She immediately experienced pain and swelling over the left knee and was not able to bear weight on the left leg. Pain on the Numerical Pain Rating Scale (NPRS) was 10/10. The pain was sudden in onset and dull aching type. The patient directly came to the hospital and was advised an X-ray. After an X-ray examination, she was diagnosed with a comminuted patella fracture (left side). She was advised primary care and was admitted with an above-knee cylindrical slab application. Two days later, on March 24, 2022, she underwent an open reduction internal fixation (ORIF) with tension band wiring under spinal anesthesia. Pre and postoperative radiological images of the patellar fracture are shown in Figure 1 and Figure 2.

After surgery, she was referred to the physiotherapy department for further management four days later, on March 28, 2022. Physiotherapy was started for manifestations such as pain, restricted range of motion, and reduced strength and to make the patient independent in her activities of daily living.

Clinical findings

The physical examination was carried out after the patient gave her informed consent. On general examination, the patient was awake, cooperative, and well-oriented in terms of time, place, and person. The patient was a known case of hypertension and diabetes mellitus (Type 2) for four years and she had a history of stroke (right side) four months earlier. On local examination, the pain was 8/10 on NPRS, dull aching type, and the pain aggravated on movement and relieved on rest. The patient was examined in a supine lying position. On inspection, a scar was present 5 cm in length over the anterior side of the left knee. Diffuse swelling was present. On palpation, tenderness (Grade 3) was present. Muscle strength was grade 1 for knee flexors.

Physiotherapy interventions

Physiotherapy interventions were aimed at reducing pain, increasing ROM to functional range, improving the strength of upper and lower limb muscles, and making the patient functionally independent for her activities of daily living. The patient's and physiotherapy management's goals were met according to the custom-made protocol. A four-week comprehensive intervention was designed to encourage long-term commitment to health-promoting practices. The inpatient rehabilitation program emphasized pain relief, increased range of motion, muscular strengthening, and early ambulation. 

Phase 1 (Weeks 1 to 2)

**Figure 3:**
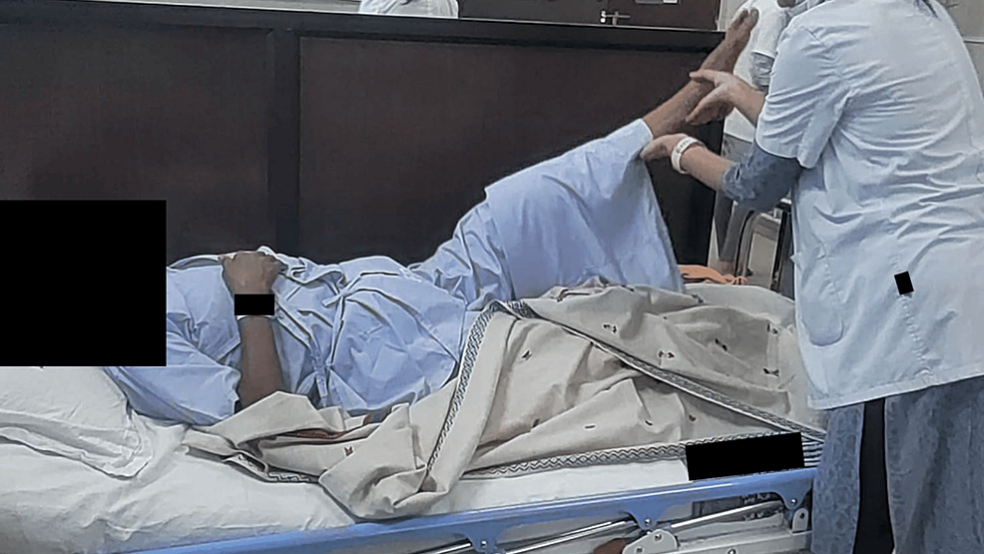
Straight leg raise exercise The therapist teaching the patient straight leg raise on the unaffected extremity

The patient was educated about her condition. Wound inspection was done and sutures were inspected. Cryotherapy with the help of an ice pack was given for two minutes for pain relief. Active range of motion to the left knee in the supine position was performed in a pain-free range. Isotonic ankle toe movement exercises for 10 repetitions were given. The patient was advised limb elevation to prevent edema (Figure 3). Isometric exercises for hamstrings were given with a 10-second hold for 10 repetitions. The patient was taught active straight leg raise. Later on, the patient was taken to sit on the bedside seat for 10 minutes. In the second week, partial weight bearing with the help of a walker was started with support.

Phase 2 (Weeks 2 to 4)

In this phase, strengthening exercises were started. Resisted exercises of the hamstrings were started. The patient was taken to the bedside seat for five minutes and then made to stand with support. The patient was advised full weight bearing with the help of a walker. Ambulation with the help of a walker was started, thereby making the patient functionally independent. A comprehensive home exercise program was taught to the patient along with ergonomic advice. The knee ROM was noted, as in Table 1.

**Table 1:** Knee range of motion assessed for four weeks. The range of motion shows improvement in the flexion ranges.

Left Knee Range of Motion	Day 1	Week 1	Week 2	Week 3	Week 4
Flexion	8	15	20	30	40
Extension	0	0	0	0	0

Outcome measures

After each week the outcome measures were recorded for four weeks. The outcome measures taken were the NPRS and the Lower Extremity Functional Scale (LEFS), shown in Table 2.

**Table 2:** Outcome measures

Outcome Measures	Day 1	Week 1	Week 2	Week 3	Week 4
Numerical Pain Rating Scale	10/10	8/10	6/10	5/10	3/10
Lower extremity functional scale (LEFS)	20/80	35/80	42/80	56/80	62/80

## Discussion

This case report demonstrates that patients with comminuted patella fractures treated with ORIF and tension band wiring have a good probability of recovery. At the end of the rehabilitation, the patient had reduced pain, increased ROM, and full weight bearing with ambulation with the help of a walker. Patella fractures are infrequent, accounting for just 0.5-1.5% of all bone injuries, and males are more commonly injured than women. Due to the nature of the trauma that generated the patella fracture, comminuted patella fractures and related fractures of other joints can also be observed [7].

The Arbeitsgemeinschaft fuer Osteosynthesefragen (AO) popularised anterior tension band concepts for patella fracture repair in the 1950s. Its stability was confirmed in subsequent experiments. Internal fixation using tension bands and Kirschner (K) wires or cannulated screws, lag screw fixation, and partial patellectomy are all modern therapeutic options with good clinical results [8]. Patients were administered a straight knee immobilizer for a minimum of six weeks after ORIF. Long-term immobilization can have negative repercussions such as reduced knee ROM and immobilized muscle weakening. These impairments result in difficulty performing activities of daily living, activity limits, and a worse quality of life.

Physical therapy can help address these problems and improve functional ability [9]. Following fracture repair, immediate weight-bearing as tolerated should be allowed; however, the time to begin ROM exercise has been a point of contention. To avoid fracture displacement, fractures were traditionally immobilized in full extension for up to six weeks after surgery. Early ROM exercise, on the other hand, may be done safely. We advocate examining fracture fixation stability intra-operatively to decide how much mobility may be tolerated right away. Within the first few weeks following surgery, patients can gradually increase their ROM while still wearing a hinged knee immobilizer [10].

## Conclusions

Comminuted patella fractures are rare fractures of the lower limb. Based on the patient's progress, a comprehensive lower extremity strengthening plan combining isometric workouts post surgery along with active to passive ROM exercises may be advantageous, while an additional study is needed to confirm their effectiveness. A future study might focus on the impact of various therapeutic activities on the quadriceps muscles, such as neuromuscular electrical stimulation, to boost their activity.
